# The Impact of COVID-19 Pandemic on Outpatient Visits for All-Cause and Chronic Diseases in Korea: A Nationwide Population-Based Study

**DOI:** 10.3390/ijerph19095674

**Published:** 2022-05-06

**Authors:** Boram Sim, Eun Woo Nam

**Affiliations:** 1 HIRA Research Institute, Health Insurance Review and Assessment Service (HIRA), Wonju 26465, Korea; simbr12@hira.or.kr; 2 Department of Health Administration, Graduate School, Yonsei University, Wonju 26493, Korea

**Keywords:** SARS-CoV-2, COVID-19, pandemic, chronic disease, outpatient care, access to health service

## Abstract

This study explores the impact of the coronavirus disease 2019 (COVID-19) pandemic on outpatient visits for all-cause and chronic diseases in 2020. We extracted the data of patients who visited medical institutions over the past five years (2016–2020) from nationwide claims data and measured the number of monthly outpatient visits. A negative binomial regression model was fitted to monthly outpatient visits from 2016 to 2019 to estimate the numbers of 2020. The number of all-cause outpatient visits in 2020 was 12% lower than expected. However, this change was relatively stable in outpatient visits for chronic diseases, which was 2% lower than expected. Deficits in all-cause outpatient visits were observed in all months except January; however, deficits in outpatient visits for chronic diseases have rebounded since April 2020. The levels of change in healthcare utilization were observed differently among disease groups, which indicates that the impacts of the pandemic were disproportionate. This study calls for a policy response to emerging and reemerging infectious diseases, as the findings confirm that a health crisis, such as the COVID-19 pandemic, could disrupt the healthcare system. Assessing the mid-to long-term impacts of COVID-19 on healthcare utilization and health consequences will require further research.

## 1. Introduction

The coronavirus disease of 2019 (COVID-19) was first identified in December 2019 in Wuhan, China, and it rapidly spread worldwide. The World Health Organization declared the outbreak to be a pandemic in March 2020 and called for governments to take “urgent and aggressive action” to combat it [1]. However, in the early stages, as no vaccine or treatment was available, countries had to rely on several non-pharmaceutical interventions (NPIs), including the closure of schools, workplaces, and borders, as well as social distancing policies, to slow the speed of the spreading virus so that healthcare systems could handle patients [2,3]. Thus, the pandemic was expected to affect people’s health both directly and indirectly, as it changed daily life.

In this context, there are growing concerns that non-COVID-19 patients have meanwhile been neglected, as health resources have been concentrated towards addressing the COVID-19 surge [4]. Recent studies have reported that the pandemic has significantly disrupted the healthcare of non-COVID-19 patients, with canceled or delayed medical attention due to COVID-19 [5,6,7]. Moreover, some patients experience adverse health consequences owing to delayed and deferred medical care [8,9]. This situation could be particularly problematic for patients with chronic diseases who require ongoing medical attention for disease management and close follow-ups. Additionally, it is now apparent that underlying conditions or comorbidities increase the risk of severe COVID-19, thus often leading to death [10,11]. Thus, patients with chronic diseases are struggling with the “double burden of diseases” caused by existing conditions alongside COVID-19. 

In Korea, since the first COVID-19 case was detected on 20 January 2020, the total number of confirmed cases remained relatively stable for a month, with 30 cases, consisting predominantly of inbound travelers from overseas or their close contacts [12]. However, starting with the super-spreader of 18 February 2020, a large-scale infection led to the nationwide spread of the virus, thus resulting in the first wave in Korea from February to April 2020. Nevertheless, the Korean government did not implement a national lockdown, unlike many other countries, but encouraged “enhanced social distancing” from 22 March to 19 April 2020 [13]. The government recommended that citizens postpone or cancel unnecessary gatherings and travel and enforced a temporary shutdown of certain facilities that were at a higher risk of infection, such as religious, indoor sport, and entertainment facilities. It was expected that the pandemic would disrupt healthcare services in Korea. Moreover, if the health status of patients deteriorated owing to deferred care, it could result in significant social and financial burdens for the nation as a whole, since deaths from chronic diseases account for 81% of all deaths in Korea [14]. 

Thus, it is crucial to understand the changes in healthcare utilization during these unprecedented times, as it could provide information on how the government responds to the current and future crises. Especially since countries have applied different policy responses to COVID-19, evidence to compare with other countries’ results is crucial to explore the most effective and efficient approaches to responding to the pandemic, including ensuring essential care to the public. However, most studies have been conducted using data from a single hospital or specific hospitals or data collected through surveys [15,16]; these results may not represent the entire country. Therefore, this study explores how healthcare utilization was affected in 2020, using nationwide data from Korea. To this end, we investigated the changes in outpatient visits for all-cause and chronic diseases and determined the impact of the COVID-19 pandemic on it.

## 2. Materials and Methods

### 2.1. Data Source 

Data were obtained from the nationwide claims data of Korea’s Health Insurance Review and Assessment Service (HIRA). In Korea, 97% of the population is enrolled in the National Health Insurance (NHI) scheme, and the government-subsidized Medical Aid Program covers the remaining 3%. Thus, the HIRA database comprises the healthcare utilization information for the entire population of Korea. We extracted data from patients who visited medical institutions during the previous five years (January 2016 to December 2020). 

In Korea, patients can go to any medical institution, including clinics, hospitals, or general hospitals, except for tertiary hospitals. Tertiary hospitals are accessible when a patient presents a referral letter from the first-contact provider. However, the referral system does not function well, as patients prefer tertiary care hospitals regardless of the severity of their disease.

### 2.2. Study Design 

Considering that most patients with chronic diseases require regular care in an outpatient (OP) setting, we measured the number of monthly OP visits. We analyzed all-cause and chronic disease-specific OP visits separately for comparison. Chronic disease was defined as the encompassing 11 conditions that can be applied to the “chronic disease management fee” in the Korea NHI scheme. The total number of OP claims during the study period was 3,667,294,598, and chronic diseases accounted for 19.5% (714,300,274 cases) of the total claims. Hypertension was the most prevalent chronic disease (31.3%), followed by mental and behavioral disorders (16.6%), diabetes (16.0%), neurological disease (8.0%), malignant neoplasm (7.9%), chronic renal failure (5.4%), heart disease (4.3%), liver disease (3.6%), thyroid disease (3.5%), cerebrovascular disease (3.2%), and respiratory tuberculosis (0.2%). We performed subgroup analysis for each of the 11 chronic diseases to investigate the differences among the disease groups.

### 2.3. Outbreak of COVID-19 in Korea

Korea experienced three waves of the COVID-19 pandemic in 2020. The first occurred in mid-February and March with a peak of 909 new cases on 29 February 2020. The government brought the outbreak under control through various social distancing policies and other quarantine measures. However, the second and third waves hit the country again in August to September, and November to December, of 2020. Consequently, as of 31 December 2020, there have been 61,769 confirmed cases and 917 deaths from COVID-19 in Korea; the cumulative confirmed COVID-19 cases were 1203 per million people, and the cumulative confirmed COVID-19 deaths were 17.9 per million people. However, these figures are relatively low compared to other countries, such as the United States, the United Kingdom, and Japan (confirmed cases: 60,566, 36,533, and 1870 per million people, respectively; COVID-19 deaths: 1078, 1056, and 27 per million people, respectively) [17].

### 2.4. Statistical Analysis 

To determine whether the pandemic significantly affected healthcare utilization in 2020, we estimated the OP visits for each month in 2020 based on the pre-pandemic period data (January 2016 to December 2019). A negative binomial regression model was fitted to the monthly number of OP visits from 2016–2019. We included covariates to adjust for monthly and seasonal variations over the study period: years (2016–2019), months (January to December), and seasons (the four seasons in Korea). Furthermore, the population was used as an offset variable in the model to reflect changes in population size. It is expressed by the following equations:

E(Y_t_) = β_0_ + β_1_ Year_t_ + β_2_ Month_t_ + β_3_ Seasons_t_ + offset (ln(P_t_)) (1)

E(Y_t_) = β_0r_ + β_1_ Year_t_ + β_2_ Month_t_ + β_3_ Seasons _t_ +β_4_ impact2020 + offset (ln(P_t_)) (2)

The monthly number of OP visits in month t were estimated using model (1). If the actual monthly OP visits fall outside the 95% confidence interval of the estimates, it indicates an excess or deficit of health utilization. Moreover, to assess whether the OP visits changed significantly on the annual time scale, we further analyzed with the variable ”impact2020” using model (2). Exp (B) of the variable indicates the relative number of OP visits in 2020 compared with the estimate. All statistical analyses were performed using SAS Enterprise Guide 7.15. 

## 3. Results

### 3.1. Trends in OP Visits from 2016 to 2020

**Figure 1:**
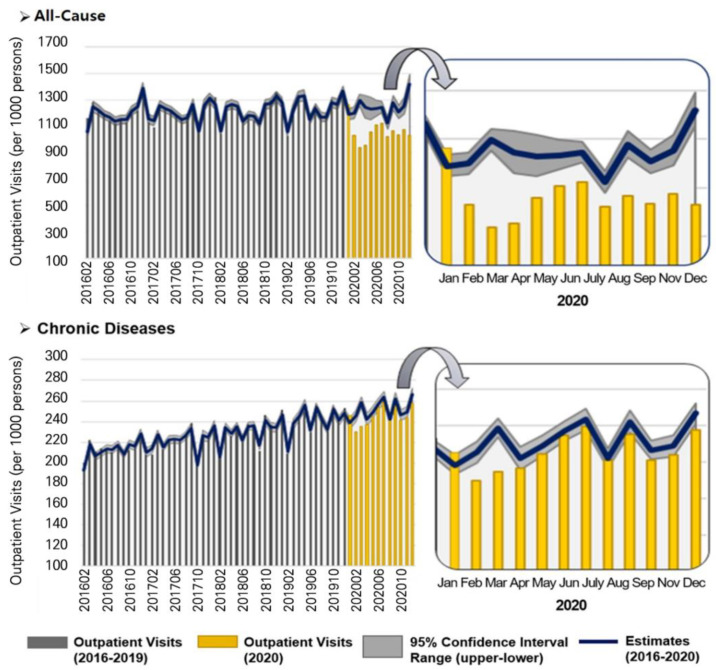
Outpatient Visits for All-cause and Chronic Disease (2016–2020). “Estimates” means the estimated outpatient visits based on the pre-pandemic period (2016–2020).

The number of all-cause OP visits declined from 1146 cases per 1000 persons in January 2016, to 1031 cases in December 2020, as shown in Figure 1. In contrast, OP visits for chronic diseases continuously increased from 204 cases per 1000 persons in January 2016 to 258 cases in December 2020 (Figure 1). 

### 3.2. Changes in OP Visits for All-Cause and Chronic Diseases in 2020 

In 2020, all-cause OP visits were 12% lower than expected (*p* < 0.001) (Table 1). Furthermore, from February to December 2020, the monthly all-cause OP visits fell below the 95% confidence interval, thus indicating deficits in healthcare utilization (Table 2) (Figure 1). Notably, when COVID-19 patients surged in number in March and April 2020, the monthly OP visits were 28% and 23% lower than expected, respectively. 

The number of OP visits for chronic diseases in 2020 was 2% lower than expected (*p* < 0.01) (Table 1). In February and March 2020, the monthly OP visits for chronic diseases were 6% and 9% lower than expected, respectively. However, it rebounded to the average number after April (Table 2) (Figure 1). 

### 3.3. Changes in OP Visits among Chronic Disease Groups in 2020

We found that, in most chronic disease groups, the number of OP visits in 2020 were lower than expected (Table 3). Whereas the discrepancies between the actual and expected OP visits were significant for respiratory tuberculosis, neurological disease, and liver diseases (11.5%, 7.1%, and 6.2%, respectively), diabetes and malignant neoplasm had smaller discrepancies (2.7% and 2.3%, respectively). In addition, heart disease, thyroid disease, and mental and behavioral disorders showed a 4% discrepancy. The actual OP visits were higher than expected for hypertension and chronic renal failure, but the difference was not statistically significant. 

Furthermore, Table 4 presents the monthly results by chronic disease groups; the monthly OP visits in February and March 2020 fell outside the 95% confidence interval for all the disease groups except for chronic renal failure, thus indicating healthcare utilization deficits. In April 2020, the figures rebounded to the previous average for hypertension, cerebrovascular diseases, malignant neoplasm, liver diseases, and chronic renal failure, while it did not for other diseases. In particular, the monthly OP visits for respiratory tuberculosis and neurological disease remained lower than those estimated since February.

## 4. Discussion

Our findings provide valuable insights for understanding how the COVID-19 pandemic has affected the utilization of healthcare in Korea. This study shows that the OP visits for all-cause and chronic diseases were lower than expected in 2020. The deficits in OP visits were prominent in each subsequent wave, especially in the first wave (mid-Feb to March 2020). This is consistent with a previous study in Korea that suggests healthcare avoidance was notable during the peak of the COVID-19 outbreak [18]. Several studies have reported that patients delay or avoid seeking care because they fear being infected by COVID-19 [7,19]. This fear could spread through government measures, such as social distancing. According to a public survey in Korea, the perceived risk of COVID-19 infection has fluctuated depending on the pandemic waves; the percentage of respondents who answered they were “highly likely to be infected to COVID-19” was the highest in February 2020 (19.8%), followed by December (16.8%), September (14.4%), and October (12.8%). This seems to be aligned with the changes in the OP visits in this study, indicating that the perceived risk affects the attitude towards healthcare utilization [20]. Another factor could be the changes in the medical practice of healthcare providers; during this unprecedented time, it is recommended to avoid exposure to COVID-19 and ensure continued access to essential medication. Thus, OP visits could be delayed with longer-term medication prescriptions [21]. Finally, the availability of healthcare providers might be partially affected to temporary decline of OP visits, as medical institutions had to close temporarily if COVID-19 patient had recently visited [22].

However, we observed relatively minor changes in OP visits for all-cause and chronic diseases, compared to countries such as Singapore, China, and the United States, where medical use has decreased by 30–60% [23,24,25]. This result is explained by the fact that Korea did not implement a stringent lockdown, nor was the healthcare system overwhelmed by the COVID-19 surge [16,26]. It is also important that the Korean government has made efforts to ensure safe and reliable access to healthcare services, instead of advising that patients postpone or avoid visiting hospitals. For example, Korea designates and operates ‘National Relief Hospitals’, which treat respiratory patients in a separate place to allow general patients to visit hospitals without having to worry about virus exposure. In addition, while pandemic-induced job loss or income reduction makes it difficult for patients to afford healthcare services [27], universal health coverage (which includes the entire population of Korea) has contributed to the removal of the healthcare services’ financial barrier. 

Furthermore, this change was stable in OP visits for chronic disease compared with all-cause OP visits. Whereas the deficits for all-cause OP visits were observed in all months except January, the deficits for chronic diseases were observed only around the pandemic waves. It indicates that the pandemic has affected healthcare utilization among disease groups differently. In this context, this study further expands on our knowledge by comparing the level of change in healthcare utilization among chronic diseases. Among the 11 chronic disease groups we selected, respiratory tuberculosis showed a significant drop in OP visits in 2020 compared to the numbers that were expected. The previous study suggests that quarantine measures (e.g., wearing facial masks, keeping social distancing) have positively affected the reduction in respiratory disease [28]. Similarly, the notification of tuberculosis, reported by the medical institution to the KDCA (Korea Disease Control and Prevention Agency) when they diagnose a tuberculosis patient, was also lower than expected [29]. However, given that tuberculosis has a long incubation period, it is doubtful that quarantine measures during the pandemic had immediate preventive effects in reducing the incidence of tuberculosis. Rather, it might indicate that potential patients were less likely to visit the medical institutions, resulting in fewer reports. Therefore, the result suggests strengthening the national tuberculosis control program during the pandemic to detect potential patients at the right time. 

Meanwhile, changes in neurological diseases, which also showed a more significant decline than expected, can be interpreted in various ways, as they include a wide range of diseases from the common migraine to Alzheimer’s and Parkinson’s disease. One possible explanation is that migraine patients’ threshold for seeking care has increased and resulted in a reduction in healthcare visits with milder symptoms [30,31]; migraines account for the most considerable portion of neurological diseases. In addition, patients living with Alzheimer’s and Parkinson’s disease in long-term care facilities (LTCs) may have cut back on their hospital visits for many reasons. For example, patients’ families avoid bringing the patients to OP care due to concerns of COVID-19 infection, and the LTC facilities do not allow patients to visit other medical institutions unless emergency situations occur. At the same time, medical institutions are hesitant to provide care for patients who are at high risk of being affected by COVID-19 because OP care is discouraged for residents of LTCs [32]. 

Changes in mental and behavioral disorders have shown interesting results. There are growing concerns that the pandemic has worsened mental health, such as depression and anxiety [33,34]. The 2020 statistics of the Korean NHI showed a significant increase in medical expenses for mental and behavioral disorders from the previous year [35]. However, it should be noted that patients for mental health have been steadily increasing in recent years when there was no pandemic. In other words, when we estimated the OP visits for mental and behavioral disorders in 2020, adjusting for the increasing trend over the years, it was 4% lower than expected. This result aligns with a prior study conducted in a tertiary hospital in Korea, which captured the hesitancy to seek care among patients with anxiety and depression during the pandemic [15]. Thus, if there had been an increase in the number of new patients with mental health conditions due to COVID-19, then the healthcare utilization of existing patients might have been deferred more often. 

Finally, during the pandemic, malignant neoplasia OP visits showed a mild reduction and rebounded quickly. This result is similar to that of previous studies, which reported that severe disease patients’ use of healthcare services did not decrease during the peak period of the Middle East Respiratory Syndrome (MERS) outbreak in Korea [36]. It indicates that when the risk of an infectious disease epidemic is high, a patient compares the risk and benefit of seeking care to decide whether to use such care, and those with severe conditions are not as affected by these epidemic circumstances [37]. Therefore, this unprecedented ‘natural experiment’ shows the value of healthcare services that patients perceive. In other word, it provides an opportunity to identify low-value cares to allocate healthcare resources more rationally [38,39]; for example, healthcare services that have dropped rapidly due to the pandemic might indicate low-value care. Further studies are required to determine which services have significantly dropped without negative health consequences.

This study had certain limitations. First, it examined the overall changes during the pandemic, which could differ from the results of an individual-level analysis. Patients with chronic diseases receive monthly OP care, and the treatment interval was expected to extend further because of the pandemic; thus, the study period was insufficient to follow-up healthcare utilization at an individual level. Second, as this study includes the period up to December 2020, the findings do not reflect the recent trends from the spread of the Delta and Omicron variants. Nevertheless, it is the first study to investigate the impact of COVID-19 on healthcare utilization using nationally representative data. We will further study the mid-to long-term impacts of COVID-19 on healthcare utilization and health consequences. 

## 5. Conclusions

In this study, we attempted to estimate the impact of the COVID-19 pandemic on healthcare utilization, especially for outpatient care. The findings confirmed that a health crisis, such as the COVID-19 pandemic, could disrupt the healthcare system. Therefore, our study calls for a policy response to emerging and reemerging infectious diseases to maintain essential care. Teleconsultations, temporarily allowed in Korea during the COVID-19 pandemic, would be a good starting point. 

## Figures and Tables

**Table 1:** Assessment of the Pandemic’s Impact on OP Visits in 2020.

Diseases	Actual Visits (Cases)	Expected Visits ^†^ (Cases)	Impact of the Pandemic in 2020		
			Exp (B)	95% Confidence Interval (Upper–Lower)	*p*-Value
All-cause	658,504,435	773,530,267	0.88	0.83–0.93	<0.0001 ***
Chronic diseases	152,720,186	156,625,911	0.98	0.96–0.99	0.0074 **

^†^ Annual estimates were obtained by aggregating the estimates of all months in 2020. ** *p* < 0.01, *** *p* < 0.001.

**Table 2:** Assessment of the Pandemic’s Impact on Monthly OP visits in 2020.

Months	All-Cause				Chronic Diseases			
	Actual Visits (per 1000 People)	Expected Visits (per 1000 People)	95% CI *	Change ^†^	Actual Visits (per 1000 People)	Expected Visits (per 1000 People)	95% CI *	Change ^†^
January	1263	1190	1235–1147	△	245	238	244–233	△
February	1028	1199	1244–1155	▼	230	245	251–240	▼
March	938	1296	1342–1252	▼	235	259	264–254	▼
April	955	1245	1334–1161	▼	237	242	248–236	-
May	1057	1230	1318–1147	▼	245	249	255–243	-
June	1109	1235	1296–1176	▼	255	257	263–252	-
July	1124	1245	1289–1203	▼	260	264	269–258	-
August	1023	1123	1169–1078	▼	241	242	247–237	-
September	1067	1279	1335–1225	▼	255	262	268–256	▼
October	1033	1208	1259–1158	▼	241	247	252–242	▼
November	1074	1253	1318–1191	▼	244	249	255–243	-
December	1032	1420	1491–1353	▼	258	267	273–261	▼

* 95% confidence interval (upper–lower). ^†^ △(excess): exceeding the upper limit of 95% CI, ▼(deficit): below the lower limit of 95% CI, - (average): within the 95% CI.

**Table 3:** Assessment of the Pandemic’s Impact on OP visits in 2020 (11 chronic disease groups).

Chronic Disease Groups	Actual Visits (Cases)	Expected Visits (Cases)	Impact of the Pandemic in 2020		
			Exp (B)	95% Confidence Interval (Upper–Lower)	*p*-Value
Hypertension	47,155,146	46,907,780	1.01	0.99–1.02	0.4789
Diabetes	24,639,134	25,344,665	0.97	0.96–0.99	0.0014 **
Mental and behavioral disorders	26,958,894	28,071,403	0.96	0.94–0.98	0.0002 **
Respiratory tuberculosis	237,652	270,496	0.89	0.86–0.91	<0.0001 ***
Heart disease	6,436,864	6,752,433	0.96	0.93–0.98	<0.0001 ***
Cerebral cardiovascular disease	4,563,098	4,770,861	0.96	0.93–0.99	0.0050
Neurological disease	11,561,938	12,494,176	0.93	0.90–0.96	<0.0001 ***
Malignant neoplasm	12,179,249	12,474,396	0.98	0.96–1.00	0.0482 *
Thyroid disease	5,160,853	5,381,911	0.96	0.93–0.99	0.0103 *
Liver disease	5,182,999	5,513,492	0.94	0.90–0.97	0.0006 **
Chronic renal failure	8,644,359	8,575,289	1.01	0.99–1.02	0.2734

* *p* < 0.05, ** *p* < 0.01, *** *p* < 0.001.

**Table 4:** Assessment of the Pandemic’s Impact on Monthly OP visits in 2020 (11 chronic disease groups).

Months	Changes ^†^ in Chronic Disease Groups										
	Hypertension	Diabetes	Mental and Behavioral Disorders	Respiratory Tuberculosis	Heart Disease	Cerebral Cardiovascular Disease	Neurological Disease	Malignant Neoplasm	Thyroid Disease	Liver Disease	Chronic Renal Failure
January	△	△	-	-	-	△	△	△	-	-	△
February	▼	▼	▼	▼	▼	▼	▼	▼	▼	▼	-
March	▼	▼	▼	▼	▼	▼	▼	▼	▼	▼	▼
April	-	▼	▼	▼	▼	-	▼	-	▼	-	-
May	-	-	▼	▼	▼	▼	▼	▼	-	-	-
June	-	-	▼	▼	-	-	▼	-	-	-	-
July	-	-	▼	▼	-	-	▼	-	-	▼	△
August	△	-	-	▼	-	-	▼	-	-	▼	-
September	-	▼	▼	▼	▼	▼	▼	▼	▼	▼	-
October	-	▼	▼	▼	▼	▼	▼	-	-	▼	△
November	-	▼	-	▼	▼	-	▼	-	-	-	-
December	-	▼	▼	▼	▼	▼	▼	-	▼	▼	-

^†^ △(excess): exceeding the upper limit of 95% CI, ▼(deficit): below the lower limit of 95% CI, - (average): within the 95% CI.

## Data Availability

The datasets used and/or analyzed in this study are available from the corresponding author on reasonable request.
